# Une variante rare de la fracture de Monteggia chez un adulte

**DOI:** 10.11604/pamj.2015.21.8.6802

**Published:** 2015-05-05

**Authors:** Redouane Ouakrim, Mohamed Saleh Berrada

**Affiliations:** 1 Service de Chirurgie Orthopédique et Traumatologique CHU Ibn Sina, Rabat, Maroc

**Keywords:** fracture de Monteggia, Bado IV, réduction, Monteggia fracture, Bado IV, reduction

## Image en medicine

La fracture luxation de Monteggia de type IV, selon la classification de Bado, est une lésion rare. Elle est définit par la présence d'une fracture des deux os de l'avant bras avec une luxation antérieure de la tête radiale. Son traitement est très exigeant en matière de réduction et de restauration de la longueur des deux os du cadre antébrachiale condition sans laquelle la réduction de la luxation de la tête radiale devient impossible ce qui va compromettre la fonction de la prono-suppination de l'avant-bras. Nous rapportons le cas d'un homme de 32 ans qui s'est présenté aux urgences chirurgicales pour une fracture de Monteggia correspondant au type IV selon la classification de Bado. Un traitement chirurgical par deux plaques type DCP (dynamic compression plate) a permis d'obtenir une réduction de la tête radiale automatiquement après le rétablissement de la longueur des deux os du cadre antébrachial. Une immobilisation par une attelle brachio-antébrachio-palmaire a été réalisée pendant trois semaines suivies d'une rééducation.

**Figure 1:**
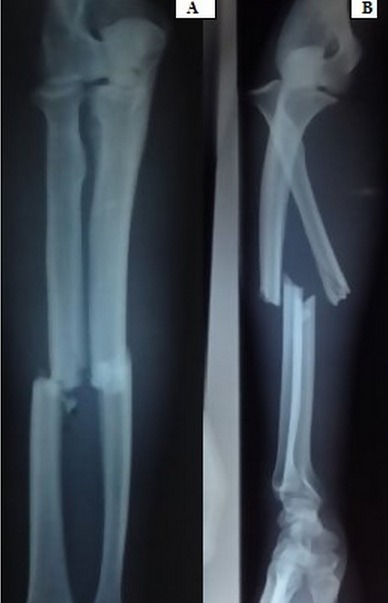
Fracture de Montegia type IV de Bado

